# In plain view: Gender in the work of women healthcare chaplains

**DOI:** 10.1177/00377686221105770

**Published:** 2022-06-29

**Authors:** Sonya Sharma, Sheryl Reimer-Kirkham

**Affiliations:** University College London, UK; Trinity Western University, Canada

**Keywords:** chaplaincy, gender, healthcare, racialisation, religion, aumônerie, genre, racialisation, religion, soins de santé

## Abstract

In sociological studies of religion and chaplaincy, there is little research on how gender plays a role in structural inequalities and experiences of women chaplains. Through research on the work of women chaplains in public healthcare in Vancouver (Canada) and London (England) this qualitative study revealed that while they have opportunities for leadership and ministry in chaplaincy, they are often on the margins of the religious institutions they are affiliated with and the secular medical organisations that employ them. Simultaneously, they confront the social structuring of gender and race that can affect them being overlooked. By applying a lived religion and feminist intersectional analysis, this research focuses on an area of study that has received scant attention.

## Résumé

Dans les études sociologiques de la religion et de l’aumônerie, il y a peu de recherches sur la façon dont le genre joue un rôle dans les inégalités structurelles et les expériences des femmes aumônières. Grâce à une recherche sur le travail des femmes aumônières dans les services de santé publique à Vancouver (Canada) et à Londres (Angleterre), cette étude qualitative a révélé que si elles ont des possibilités de leadership et de ministère dans l’aumônerie, les femmes aumônières sont souvent en marge des institutions religieuses auxquelles elles sont affiliées et des organisations médicales laïques qui les emploient. Simultanément, elles se confrontent à la structuration sociale du genre et de la race qui peut les affecter en les laissant de côté. Grâce à une analyse de la religion vécue et à une analyse intersectionnelle féministe, cette recherche se concentre sur un domaine d’étude qui a reçu peu d’attention jusqu’ici.

## Introduction

Women chaplains work in a range of contexts, but rarely are their experiences foregrounded. Within healthcare, they provide vital support to staff, patients, and families facing fragility of life and illness. They are often found in public healthcare – a microcosm of society that reflects a complex social conjuncture of religious and cultural plurality, social inequalities, and political and economic change. In this context, they simultaneously confront questions about their legitimacy because of financial pressures on healthcare systems, secularising societies, and the structuring of gender and race that can affect them being overlooked.

Drawing on qualitative research in public healthcare in London, England and Vancouver, Canada, we elucidate accounts of women chaplains, demonstrating how gender and racialisation play a role in the visibility of their work. We shed light on how healthcare chaplaincy offers women roles of responsibility and leadership despite them being on the margins of the religious institutions they are affiliated with and the secular medical organisations that employ them. By applying a lived religion and feminist intersectional analysis, our research reveals the significance of gender in chaplaincy, an area of study that has received scant attention within sociological study of religion and chaplaincy.

## Gender as obscured in chaplaincy research

Recent literature reviews (Pesut et al., 2016; Timmins et al., 2018) report that the chaplaincy literature focuses on the experiences of chaplains and their role on the healthcare team, the perceptions of patients regarding chaplaincy care, and outcomes of their care. Analyses of gender and women chaplains’ work is absent in the articles included in these two reviews. Subsequently, we conducted a literature search on the intersecting concepts of gender (women), chaplaincy (spiritual care providers), and healthcare in CINAHL Complete, Academic Search Complete, Atla Religion Database with AtlaSerials PLUS, MEDLINE, OpenDissertations, APA PsycArticles, APA PsycInfo, and SCOPUS databases for 2015–2021, which yielded two related articles on gender and chaplaincy in the United States (White et al., 2021a, 2021b). These articles focus on demographic diversity among chaplains, educators, and trainees and the professionalisation of female and male chaplains. As such, there is little research on women healthcare chaplains and the gendered nature of their experience. More research is needed on gender, non/religious, and racial inequalities in chaplaincy (Bryant, 2018; Hutt, 2019; O’Donoghue, 2020).

Several factors contribute to the absence of analyses of gender and women chaplains. First, men continue to outnumber them. For example, in England, ordained women chaplains make up 32% of which 45% work in healthcare (Church of England, 2020: 3, 48). In the United States, across sectors, 30% are women and largely Mainline Protestant (White et al., 2021a; Zippia, 2020). The gender imbalance towards men in chaplaincy means it is not unusual to have research samples with more men, and hence findings that privilege male experience. Although more women have become chaplains since the late mid-twentieth century, they are not typically at the centre of research. For example, in Hansen’s study, when quoting participants, he ‘refers to all [military] chaplains as male in order to protect chaplains’ anonymity’, but this risks women chaplains becoming invisible (2012: xiii). In a US study on port chaplains, 4 out of 21 were women (Cadge and Skaggs, 2019). Of 17 Muslim chaplains in research on prison chaplaincy in Canada, England, and Wales, two were women (Beckford and Cairns, 2015). In UK universities, of the 367 chaplains who took part in interview research, 37% were women (Aune et al., 2019). Similarly, in a study on prayer in healthcare in Canada and England, 40% of chaplains were women (Reimer-Kirkham et al., 2020). Male chaplains may thus be more researched because they are more numerous, which can result in women’s experiences and gender as a structural experience within this profession side-lined.

Second, a recurrent theme in this literature that may inadvertently obscure gender analyses is the liminal position that most chaplains occupy, regardless of gender. This theme sheds light on religion’s place in secularising societies. Kühle and Christensen’s (2019) research on Lutheran chaplains in the Danish military, hospitals, and prisons found that similar to Sullivan’s (2014) sample of American chaplains, they offered a ‘ministry of presence’ to provide for all faiths, bridging religious differences and the religious and secular. In research on religion in healthcare in the United States and Canada, chaplains traversed positions between medicine and religion, pastoral and clinical staff, and religion and nonreligion (Cadge, 2012; Reimer-Kirkham et al., 2020). This can result in chaplains navigating liminality in their roles or what Davie (2015) has described as ‘ambiguity’. Norwood’s (2006) study of American chaplains in a modern-day hospital revealed that they negotiated their place on the margins, learning a new language of hospital medicine to fit in and minimise their difference and unequal status (also see De Vries et al., 2008; Swift, 2016). Chaplains also navigate paid and unpaid roles. Chaplaincy or spiritual care services in state-run organisations often battle fiscal constraint, depending on how important the state deems them. In Canada’s public healthcare system, a whole chaplaincy team was made redundant (Timmins et al., 2018). In the Netherlands, ‘the capacity for spiritual care in prisons has been reduced since 2008 to almost 50 percent’ (Vellenga and De Groot, 2019: 230). Chaplaincy teams thus often run on a bank of volunteers, affecting their status and visibility (e.g. O’Donoghue, 2020; Reimer-Kirkham et al., 2020). Liminality, working between the edges, though could be an asset. University chaplains who are ‘at the edges of these institutions, are also bridge-builders to a diverse range of groups’ (Aune et al., 2019: 10). Male clergy were attracted to healthcare chaplaincy because they could work ‘on the edge’ of ‘something central to life’ versus ‘something peripheral’ (Hancocks et al., 2008: 176). Despite their liminality, the professionalisation of chaplaincy has given chaplains a place in secular organisations. Yet, gender has not been a point of examination in these studies.

Third, where diversity and inclusion have been the feature of chaplaincy research, the common phenomenon of investigation has been religious diversity and multi-faith chaplaincy, not gender. Scholars have noted tensions between accommodating religious diversity and historical Catholic clout in Spanish prisons (Martínez-Ariño et al., 2015), and in Dutch prisons Muslim chaplains managing a state system watchful of radicalisation by providing for all faiths (Vellenga and De Groot, 2019). Eccles (2014) found that an Anglican Christian hospital chaplaincy in Northern England had become more multi-faith and person-centred in its approach because of its diverse constituency, while O’Donoghue (2020) reported that the nonreligious are inadequately provided for in healthcare chaplaincy in England. Diversity and inclusion are important concerns because they raise issues of ethnic and (non)religious inequalities. However, gender and women’s experiences are largely missing in this research (Bryant, 2018).

The tensions and positions chaplains navigate have been magnified because of what they can reveal about religious change. These religious vantage points in secular organisations are what Cadge and Konieczny (2014) describe as ‘hidden in plain sight’. They argue that the consideration of religion within secular organisations be on a level with how scholars of gender and race helped to normalise these as structural and individual experiences in the workplace. While this important work is being done through research on chaplains, examining religion as a lived and intersecting experience with gender and other structures of advantage and disadvantage can push sociological studies of religion and chaplaincy forward. This research on the work of women healthcare chaplains brings the lived intersection of religion, gender, and race to the foreground. Often obscured by their male peers and wider chaplaincy membership, it brings their gendered experiences into view.

## Theoretical framework and methods

### Theoretical framework

This lacuna led us to examine how gender plays a role in structuring women healthcare chaplains’ work experiences, and how the women challenge this social structuring. We applied a lived religion and feminist intersectional theoretical framework to our research. Lived religion as a mode of analysis allows one to examine the everyday practices of religion that are away from official representatives and settings (Hall, 1997). It considers the ordinary and mundane such as acts of prayer while gardening or visiting a roadside memorial. This mode of analysis, however, has been critiqued for creating a binary between the official and unofficial, as if lived religion does not happen in institutional settings, among formal religious gatherings, or by official spokespersons, when really it does and as was evident among our participants (Ammerman, 2016). In healthcare contexts, religion traverses the official and the unofficial. For instance, prayer for a patient in an elevator before surgery, the Holy Eucharist given at the bedside, or a collective gathering in the Muslim prayer room for *jummah* or Friday prayers (Reimer-Kirkham et al., 2020). Among participants, religion was lived in informal and formal ways and spaces and implicated in the power relations and social structures that converge with them.

Lived religion as a mode of analysis complements a feminist intersectional analysis as it can reveal the suppressed realities and stories of those not typically at the centre. These standpoints similarly ask, ‘what more there is to religion: whose lives, experiences and associational forms are being overlooked?’ (Woodhead, 2013: 11). Women’s experiences of gender are constituted in relation to other social categories and structures, including race and religion, that can cause them to be rendered invisible. Legal scholar Crenshaw (1989) conceptualised the term ‘intersectionality’ to help make these intersections discernible. Her work builds on the seminal work of Black women scholars, writers, and activists who were theorising interconnections of gender, race, and class in women’s lives (see Anna Julia Cooper, Sojourner Truth, the Combahee River Collective, Gloria Anzaldúa, Patricia Hill Collins and Audre Lorde). Crenshaw (2016) states that intersectionality ‘is not primarily about identity. It’s about how structures make certain identities the consequence of and the vehicle for vulnerability’ (n.p.). Intersectionality enables examination of the operatives of oppression and advantage, which can work together to include on some accounts while excluding on others. It assists in understanding ‘differences within differences’ (Yuval-Davis, 2006).

### Methods

Our research was based in London, England, and Vancouver, Canada. London is known for its arts and culture, architecture, and public parks alongside historical class divisions and a changing demographic. The Church of England still has social, state, and institutional influence as is evident in our participants’ worklives but increased secularisation and shifting social mores have impacted Anglican affiliation from 40% in 1983 to 12% in 2018 (Voas and Bruce, 2019: 20). According to the UK Census 2011, in London, ‘48% identified as Christian, 21% as no religion, 12% as Muslim, 10% as Other and 9% not stated’ (Office for National Statistics (ONS), 2012: 5). Vancouver is known for its proximity to nature, culinary scene, and expensive housing amid deprivation and Canada’s long overdue reconciliation with its Indigenous peoples. In Vancouver 42% identified as Christian, 41% with no religious affiliation, and 17% identified as Sikh, Buddhist, Muslim, Hindu, Jewish, Traditional (Aboriginal) Spirituality, and Other religions (Statistics Canada, 2011). Secularisation, the rise of individualised and ecological spiritualities, and increased religious diversity due to migration affect Vancouver’s religious landscape. Both cities have public healthcare systems where this complexity dwells.

Women chaplains’ data are drawn from two qualitative projects. The first investigated expressions of prayer in 21 healthcare sites in London, and Vancouver (2015–2018). Of the 32 chaplains that made up the larger sample of participants, from London there were 7 women and from Vancouver there were 6. From this project it became clear that gender was operating in the background but not operationalised as one of our overarching themes of analysis. We therefore developed a study to focus specifically on gender in the work of women healthcare chaplains (2019–2020). We examined how they encountered and challenged institutional power structures and in turn how these structures impacted on their religious and spiritual and work trajectories. The sample size for the follow-up project was 12 women from 9 sites. Three were based in Vancouver and nine in London. The total sample size of women chaplains is *n* = 25.

All participants identified as women and were recruited for heterogeneity in ages, non/religion, and ethnicity. They comprise paid chaplains (full and part-time) and unpaid spiritual care volunteers. We include both groups as they were carrying out similar roles and tasks, although we were aware that time afforded to the role and responsibility differed, such as in relationship to leadership. Several spiritual care volunteers had been volunteering for years, in one case 30 years, and had become vital members of the chaplaincy team. Volunteers were more prominent in London than Vancouver where chaplains were mainly paid. While titles and levels of responsibility differed, we use the title of ‘women healthcare chaplain’ in our research to convey the context and their identification with the chaplaincy teams in which they worked. We also recognise the tensions posed by nationality and ethnicity identifiers and protecting participants’ identities.

Before our research proceeded, we obtained ethical approval from University and Health Authority Research Ethics Boards. The first project employed a series of interviews, including walking interviews to examine the spaces in which chaplains worked and how they understood their roles. Walking interviews enabled participants to lead and show us their daily routine spaces while reflecting on challenges and opportunities that occurred in their work (Clark, 2010). The second project applied biographical interviews ‘that are rich in detail but also experientially inclusive and reflexive in character’, using open-ended questions to understand the women’s life stories (Merrill and West, 2009: 113–114). Walking and biographical interviews helped to capture ‘religion-as-lived’ (McGuire, 2008). At the end of the second project, we conducted a group dialogue with participants and invited scholars to explore the women’s work amid social and religious change. Qualitative thematic data analysis evolved from close reading of the interview transcripts to the development of code categories (e.g. gender, inclusivity, religious institutions) to the final construction of overarching themes. From these emerged specific strands focused on here: choosing of chaplaincy because of more equality and inclusion, and gendered and racialised experiences of being a chaplain.

## Choosing chaplaincy: more equality and inclusion

The theme of choosing chaplaincy emerged from our analysis of how the women came to their role and reasons for taking up this profession. For several, it was the ability to minister and enact their faith in ways not typically available to them in their religious institutions. This is largely because of historical patriarchal structures that posit men in leadership at both lay and official levels. Religious women have long-pushed back for more equality. Christian women have done this throughout the feminist movements in the West, contesting androcentric interpretations of scripture, leadership, and women’s subservience (e.g. Daly, 1985; Fiorenza, 1992; Higginbotham, 1993). Muslim women also have a history of feminist activism bringing to light issues of patriarchy and oppression in Islam such as in how religious texts, leadership, and spaces are interpreted, assigned, and allocated (e.g. Ahmed, 1982; El Saadawi, 2007; Lewicki and O’Toole, 2017). This feminist history and activism operated in the background of our participants’ stories with more equality and inclusion, contributing to their choices to become chaplains.

Maryanne of European heritage had been working as a Christian healthcare chaplain for 15 years. She explained that both she and her husband had gone to university and subsequently trained to become ordained ministers in the Church of England:

While both of us trained full-time for full-time parishes [it is assumed] that the woman becomes her husband’s unpaid curate. I didn’t want to do that, and neither was it particularly easy to live without being paid (…) [At the end of my curacy] I got the attitude from various church people, officials and so forth, ‘your marriage is unfortunate’. Because we were married, they said [to me] ‘you’d be lucky to get anything paid’. Having worked hard as a curate for five years that was not great (…) When ordination came through for women (1994), not everybody agreed with it. Some Bishops didn’t, and to start the process [of ordination] you must be sponsored by a Bishop.

In the Church of England, women could finally be ordained priests in 1994, but not bishops until 2014. Curacy, as Maryanne mentioned, is the period of ministerial training after one has achieved their ordination in the Church of England. It was assumed that because she was married, she would be in a continued state of training, a static position, because of how gender roles have been perceived in the Church. While circumstances have improved for women in the Church of England, ‘In 2019, of the total number of ordained ministers, 32% were women and 68% were men’ (Church of England, 2020: 33). There has been movement, but not much. Walter and Davie contend that ‘Once inside the church, women may find themselves deprived of status and power vis-a-vis male members’ (1998: 645) and a lack of status within the Church is indeed one reason women may leave. Greene and Robbins argue that the ‘Church of England is a gendered organization and while women confront sex discrimination, they at the same time experience satisfaction with their work despite the difficulties’ (2015: 405). Although Maryanne did not leave the Church, she questioned the patriarchal order of leadership and how she could utilise the skills she had worked so hard to obtain. Chaplaincy was a way to do that. She was ‘fortunate’ to get a part-time job as a chaplain at a local hospital, which she stitched together with another part-time chaplaincy post at a nearby hospital. Chaplaincy allowed her to live out her religious ordination. Ending her interview, Maryanne said she was inspired by the suffragettes to keep ‘fighting, fighting, fighting’ for equality in the Church.

As society has moved forward in relation to wide acceptance of LGBTQ (lesbian, gay, bisexual, transgender and queer) persons and rights, and women as leaders common in many fields, the Church of England has moved little (Greene and Robbins, 2015). In contrast, chaplaincy presented Maryanne and others a space of possibility and recognition that mirror the changing values of society (Hancocks et al., 2008). Kate who was also of European heritage and ordained in the Church of England explained how chaplaincy had provided a space for her:

I went into chaplaincy at a time I’d begun to feel alienation from the Church institutionally or the authorities of the Church (…) I think people who have sexual orientation variance or gender variance, which is myself, gender-variant, then healthcare chaplaincy has been hospitable, because the hospitals are governed by the Equality Act and the protection of special characteristics, whereas some of our churches, the Church of England being one of them and I’m Church of England, do not have full equality around these and it can be quite precarious for LGBTQ people working in parishes. They often feel safer in hospitals. But it’s not totally safe.

In England, because hospitals are secular publicly funded spaces, they are required to uphold the Equality Act 2010 (Equality and Human Rights Commission, 2014) which contains several protected characteristics, including gender reassignment, sex and sexual orientation, and religion and belief. The Church of England has its own legislation that does not easily fit in with the legislation for the secular sphere. Women clergy, for example, are not afforded protection from anti-discrimination legislation within the Equality Act 2010 (Greene and Robbins, 2015). Because of this, Greene and Robbins who draw on the work of feminist scholar Acker, ‘argue that the Church of England is an example of a contemporary “inequality regime”’ (Acker, 2006: 443 cited in Greene and Robbins, 2015: 408). Acker (2006) explains that inequality regime is an analytical approach that aims to uncover the complexity and reproduction of inequalities in workplaces. While the healthcare settings in which Maryanne and Kate worked were not without structural gender inequalities (e.g. gender pay gap), they and our participants confirmed that chaplaincy was a space of inclusion in so far that it provided a space for women and gender-variant clergy to live out their religion, carry out ministry, and to progress professionally and personally.

Muslim women who had taken up the role of healthcare chaplain had similar experiences and played significant roles on chaplaincy teams. Amara was of Arab heritage and paid part-time for her work. She had been in the role for 10 years. Nadira was of Afro-Caribbean heritage and had been a ‘chaplain volunteer’ for 5 years. Nadira told us how she came to the role:

The imam asked me to come and give them support because there were no [Muslim] women to help Muslim women (…) Sometimes the men ask you to pray for them and maybe touch them on their head, which I used to find strange in the beginning because Muslim men, they’re so conservative, they won’t say that you’re an imam, they’ll say ‘women are not imams’ but that’s their thinking because the word imam means leader, which they have assigned to [men]. They can’t see that a woman is an imam (…) A man asked me to touch his head. It didn’t matter to him when he is sick, if I was a man or if I was a woman but I’m doing the prayer and that’s what he needs. He probably wouldn’t talk to me outside the hospital, but in that moment [he did].

Nadira enacted forms of leadership and ministry that she might not have been able to do in her community because of the frequent exclusion of women on governing boards of local mosques and in ritual leadership (Lewicki and O’Toole, 2017). Lewicki and O’Toole (2017) have addressed in their research on Muslim women’s activism in the United Kingdom how they enter into acts of ‘undoing’. By reinterpreting who was a leader in Islam and touching a male patient’s head after a prayer, Nadira expressed the undoing of expected norms in two ways. First, she acted counter to mainstream stereotypes that typically posit Muslim women as silent and concealed. Second, she enacted ‘an undoing of established Islamic practices that enable an enacting and ritualizing of a more inclusive Islam’ (Lewicki and O’Toole, 2017: 164). The hospital chaplaincy context in which Nadira worked helped to foster this ethos. She said, ‘it’s egalitarian in the work that we do here’. However, her role was unpaid in comparison to her fellow male imam. This may be related to gender bias insofar as male imams represent the community more prominently and are therefore hired, and fiscal constraints that healthcare chaplaincy teams often confront mean paid positions are not representative (Gilliat-Ray et al., 2013).

Nadira’s acts of care reflected the inclusive multi-faith secular institution in which she worked and her own approach to Islam. As part of the chaplaincy team, she was included because it was not assumed that a religiously different female or male chaplain could simply do her work. She did not limit her chaplaincy work to her faith group but ministered to those of other faiths too. These everyday acts of faith and inclusion can go unnoticed and remain on the margins. But her very presence alone was central to helping patients through difficult moments and to slowly unravelling the boundedness of conventional gender expectations with religion. Amara explained that her inclusion on her chaplaincy team had been vital, especially to Muslim women patients:

Due to segregation and cultural practices, Muslim women are very ‘grateful’ for the services of a female Muslim Chaplain. At a highly emotional time it is recognised that an imam might not be able to physically comfort a Muslim female patient, mother, relative or friend. [The women] would not be able to offload emotional or personal issues affecting them as with a female chaplain. Two weeks ago, I met an elderly patient whose daughter told me that her mother felt very uncomfortable when the [male] imam approached her.

Both Nadira and Amara positioned themselves as ‘knowledgeable female religious subjects’ (Bracke, 2008: 193). By doing so they disrupted traditional gendered hierarchal norms that can be found within Islam. Muslim women chaplains could be seen as leaders among the larger *ummah* or global community of Muslims who visit or use the hospital even though many both in and outside of Islam may not recognise this. Similarly, Christian women chaplains could be seen doing this. They transgressed the traditional expectations of male leadership that can be found within Christian ministry by becoming ordained ministers and chaplains. Feminist and critical race scholar, hooks, defines the margin(s) as ‘a site of radical possibility’ (1989: 20). For women in this research, while their faith traditions could marginalise them, it is on the edges of their religious institutions in healthcare chaplaincy that they found a space of their choosing, where they could live their religion and be nourished to venture out, become, and undo.

## Gendered and racialised experiences

The women worked in and from the margins in different ways. For some, becoming more visible in their role was related to more recognition in their position as a chaplain or expanding their role as a leader. For others, it was working with the challenges of gendered and racialisation of religion and spirituality which could render them as under-represented and marginalised. In employing the term ‘gendered’ we are using it to denote conventional interpretations of femininity that the women encountered. On gender and race, Crenshaw (1989) notes the unmarked structural advantages of Whiteness when gender becomes the single axis by which White women’s experiences of disadvantage in the workplace are examined. She emphasises that in contrast to the unexamined advantages of Whiteness, for Black women and women of colour, gender and race cannot be examined separately. Race as marked and unmarked is part of the process of racialisation. Racialisation is an outcome of how people are categorised and viewed and becomes meaningful through socio-cultural and psychological processes (Fanon, 2008 [1967]; Phoenix, 2006). It involves ‘ascribing sets of characteristics viewed as inherent to members of a group because of their physical and cultural traits that can include skin tone or pigmentation, and language, clothing, and religious practices’ (Garner and Selod, 2015: 12). In this section we address the theme of gendered and racialised experiences of being a healthcare chaplain – how some of the women experienced the structural disadvantages of their gender but not their race, revealing the workings of Whiteness, and others both. This social structuring affected their lived religious, spiritual roles and practices.

In our thematic analysis, essentialised constructions of gender, namely of women as caring and men as leading, operated in the day-to-day visibility and practice of chaplaincy. The women were aware of the gendered nature of caring and working in healthcare contexts that could be stratified by gender: Mavis, a Christian chaplain of Afro-Caribbean heritage, said that in her hospital experience ‘most of the doctors are men, most of the nurses are women’, while others noted several women as doctors and senior administrators. The social contexts of the healthcare settings in which the women worked were imbued with power relations concurrently mediated by politics of gender, race, and religion (Collins and Bilge, 2016). In our analysis, we observed that the women challenged notions of fixed identities of gender that place religious women’s work behind the scenes, out of the centre. May who was an Asian Catholic Sister and had worked in chaplaincy for 4 years, explained how it worked between her and a male priest: ‘Priests have authority… priests and sisters go together but I have to step back because I don’t have that authority… that challenged me; why not a woman?’ She also discussed the challenge of being a volunteer chaplain as opposed to one who was paid. She said, ‘As a volunteer I felt limited but [now] being a [paid] chaplain, I can get involved more, I’m not in the background’. Being paid affected her position and contribution. It gave her more equal status and a stronger sense of membership of the chaplaincy team. Research done by sociologists of religion on gender have long noted how religious women often negotiate relations of power and positions (e.g. Brasher, 1998). The women challenged essentialised constructions of gender in their roles as chaplains by living out ‘multiple subjectivities… having agency about who they chose to be’ (Collins and Bilge, 2016: 125). The role of chaplain within a secular context provided them a space in which to do this. Esther, a Catholic chaplain of European heritage who had been working as a chaplain for 5 years described how she had grown in her ‘priestliness’, even though her denomination does not allow women’s ordination. ‘I felt this weight of authority on me, and it felt priestly. That caused a disruption in my life because it felt right, completely [me], and yet what the [Catholic] Church said about women was not that’. Women are not able to be priests in her religious tradition because of the historical project of patriarchy that upholds male leadership. In healthcare chaplaincy she and others enacted their agency to do things differently, taking up opportunities to expand their skills and push the bounds of religious norms for their own religious flourishing and visibility.

The women explained that while they had made headway into roles of responsibility in chaplaincy, they were under-represented as leaders. Ruth, a chaplain of European heritage and ‘a wide-open Christian stance’, described her observations of leadership within clinical institutions and chaplaincy training:

At high level meetings, it’s other men and me. That was part of the landscape and still largely is. It’s been interesting finding my voice around those tables (…) You’ll hear people talk about the old boys’ club and that’s historical… I’ve heard it as recently as a few days ago.

Gender stereotypes and discrimination exist in the workplace because of ‘cultural beliefs and organizational structures, policies and practices’ (Bobbitt-Zeher, 2011: 767). These can become ‘institutionalised, as part of organisational structures that are legitimised through policies and practices, often appearing gender-neutral and formalising men’s privilege in the workplace’ (Bobbitt-Zeher, 2011: 767). Of the 25 women we interviewed, four were or had been leaders of their chaplaincy teams. They sat on committees and attended important meetings central to the running of hospitals and were aware that they needed to embody aspects of power to remain in or make themselves visible in these roles. Kate told us how clothing played a part:

The power dressing was extraordinary because you must look the part don’t you for these very powerful roles [women leaders in the hospital] and my goodness you need all the authority you can get to be taken seriously (…) it’s important to not give away your power.

Through ‘power dressing’ women chaplains could create and recreate gender (Butler, 1990) in how it is perceived in relation to religion and in medical contexts. Clothes were one way in which women chaplains in the public space of healthcare could denote weight and influence and how they lived their religion among hospital constituents. Mavis discussed how wearing a priest’s collar could convey ‘authority’ and presence among patients and staff on hospital wards. As a Black woman chaplain working in a hospital, wearing a collar meant she was regarded differently. She was not assumed to be a nurse: ‘[Without my collar] they don’t realise I’m a chaplain, they think I’m a nurse because they don’t usually have Black chaplains’. Nursing was prominent among women migrants from the Caribbean to Britain and Canada in the mid-1900s. Wearing a collar raised her visibility, but her position was unexpected. While there have been moves to ensure chaplaincies are multi-faith, it does not always mean they are gender equal and representative of all faiths and ethnicities. Mavis pointed to this discrepancy:

When people think of the chaplaincy, they think of the Church of England which dominates because they probably see that most chaplains are White (…) so when they see [me] they don’t think [I] can represent them but that’s not true.

She highlighted how the Church of England and chaplaincy have been racialised as White, which links more broadly to how ‘Christianity has been racialized through its association with whiteness’ (Joshi, 2006: 212), emerging from colonial histories that linger today in gendered and racialised assessments and in meanings and forms of exclusion and inclusion (France-Williams, 2020). This was perceptible in other accounts: Joy a volunteer Catholic chaplain of Black African heritage had her prayers refused by an older White Catholic priest, who ‘knows men but not women’. Although he had not been overtly racist towards her, sexism and racialisation can intersect to operate covertly and therefore result in the disregard of another.

Whiteness and the Anglican and Catholic Churches have functioned in Canada and England as ‘racial and religious norms’ (Joshi, 2006: 212). Chaplaincy has often been complicit in these norms regarding leadership and team membership, with recent efforts to be more inclusive. An Indigenous woman elder in a Vancouver hospital however alluded to these norms that affected her work of the sacred. Tammy said, ‘To me the hospital administration could really look and learn about all the different sacred ways of the First Nations. They don’t really accommodate what the needs are. Even to have a smudge’. Lived aspects of Indigenous spirituality were often unacknowledged. She told us about experiences of being ignored by hospital staff in trying to reach community members who were being hospitalised and being ‘paid an honorarium and not a salary’ like the chaplains in the hospital. The chaplain role is often still read as White, Christian, male, trained and accredited. This can result in Indigenous ways of knowing being discounted, and ‘racialized and culturally distinct peoples, such as Indigenous peoples not receiving the services they require’ (Turpel-Lafond and Johnson, 2020: 6). Across Canada, histories of colonialism have devastated Indigenous communities and many Indigenous women and men continue to deal with this ‘historical trauma as lived marginalization’ (Dodgson and Struthers, 2005). In the healthcare context, Tammy lived this marginalisation despite her efforts to push for visibility to help her community confronting illness and fragility of life.

Our Muslim participants experienced issues of visibility and acceptance related to gender and the racialisation of religion. Male imams are not immune to this (Spalek and Wilson, 2001). Amara explained that ‘many Muslims don’t know about Muslim chaplaincy, chaplains or Muslim female chaplains. Sometimes it’s questioned if they are needed… Staff have sometimes been prejudiced towards those of a religious background, not just Muslims’. Therefore, to make herself known to staff and Muslim patients that would like to see a Muslim chaplain she would ‘not rely on a hospital patient list but start at the top floor of the hospital wards and move her way down to make sure not to miss a Muslim patient’. She told us that it was ‘a good way to make herself known to staff as they work shifts and change a lot too’. Part of her everyday religion was to encounter those of Islamic faith. As a Muslim woman, Amara chose to wear a *hijab*. In her walking of the wards to be seen and known she lived out what Mirza (2012) has noted the tension that many Muslim women confront, which is being both visible and invisible amid anti-Muslim sentiment and normative (White) experience. Muslim women are ‘racialised through religious signifiers’ such as ‘headscarves’ (Garner and Selod, 2015: 16). Thus, ‘ethnic dress becomes interchangeable with tradition and essentialism when the female body enters the unstable arena of scrutiny and meaning’ (Mirza, 2015: 42). These everyday prejudices and exclusions mean public institutions are not always a level playing field even though more Muslim chaplains are becoming professionalised via accredited programmes that include non-Christian approaches (Gilliat-Ray et al., 2013).

Racialised women (and men) have faced exclusion but have arrived in the past decades occupying spaces in which they were once ‘constructed out’ (Puwar, 2004). Several have moved from the margins closer to the centre of chaplaincy teams. Mavis, Amara, and others have disturbed the status quo to take up ‘privileged’ positions which have not been ‘reserved’ for them, for which, they are not, in short, ‘the somatic norm’ (Puwar, 2004: 1). Puwar refers to the ‘somatic norm as the corporeal imagination of power naturalised in the body of white, male, upper/middle-class bodies’ (2001: 652). Hospitals are public spaces in which society cannot be contained but are dynamic and where those from several backgrounds cross and dwell. Healthcare chaplaincy has started to mirror such multiplicity and change in the make-up of its leadership and teams. Issues of gender and career progression have begun to be addressed. Yet, depending on hospital community demographics, racialised religious women (and men) continue to shift between margin and centre. The long association and perception of ‘chaplain’ as White, male and Christian still leaves many racialised (non)Christian female (and male) chaplains as misunderstood by staff and patients (Bryant, 2018). As Amara alluded to with her walking of the wards, it is through community building among hospital constituents that they begin to be recognised and accepted in their work and roles.

## Conclusion

Through this research, we have begun to address the lacuna that exists in the literature on women’s gendered experiences of chaplaincy. The women healthcare chaplains in this research reflected the changes to chaplaincy but also the fixed perceptions that remain in who embodies such a role and the histories that these embodiments continue to be tied to. Although they may minister to and acknowledge those of all faiths and none, it does not mean that they are treated with mutual recognition and acceptance. They constantly navigate their role between secular and sacred, science and religion, and official and unofficial. When gender is added it can make these navigations more complex with other intersecting axes making each woman chaplain’s experience different.

In London and Vancouver, the women experienced the structural constraints of their gender, religion, and race, and this could take its toll. The paradox of the women chaplains we studied was their choice to persevere and become visible in the margins of their religious institutions and secular workplaces. Amid the inequalities they confronted, they chose chaplaincy because it offered an alternative space, a space to improvise (hooks, 1989). As Esther said about taking on the role of chaplain, ‘I became more risk-taking’. Chaplaincy is where they could get the work done for others and live out their religion with their selves evolving and flourishing.

This research sheds light on the understudied area of gender and women chaplains. It has taken an intersectional approach to the study of gender, demonstrating its nexus with racialisation and other structural oppressions and inequalities. Our data collection was completed shortly before the coronavirus pandemic took hold, during which the project themes have been amplified. The pandemic revealed and deepened social inequalities, including those of gender, race, and religion (Public Health England, 2020). Religion is often perceived as lived in the private realm, but women healthcare chaplains are very much in the public space of healthcare where they confront diverse landscapes of religion, nonreligion, and spirituality. They are in plain view.
